# SEXUAL MOTIVES, SEXUAL FREQUENCY, AND SEXUAL SATISFACTION AMONG MIDDLE-AGED DIFFERENT-SEX AND SAME-SEX COUPLES

**DOI:** 10.1093/geroni/igac059.1922

**Published:** 2022-12-20

**Authors:** Hye Won Chai, Sara Mernitz, Debra Umberson

**Affiliations:** The University of Texas at Austin, Austin, Texas, United States; The University of Texas at Austin, Austin, Texas, United States; The University of Texas at Austin, Austin, Texas, United States

## Abstract

Reasons for having sex and frequency of sex are significant correlates of sexual satisfaction. However, the possible interplay between sexual motives and sexual frequency remains unexplored. Also, prior studies on sexual satisfaction largely focused on heterosexual couples and less is known about the experiences of same-sex couples. Using dyadic survey data collected from 838 middle-aged spouses in 419 gay, lesbian, and heterosexual marriages, this study examined whether the associations between sexual motives and sexual satisfaction differed by sexual frequency and whether these dynamics varied across gay, lesbian, and heterosexual couples. Results showed that intrinsic sexual motives (e.g., for enjoyment and pleasure) were associated with higher sexual satisfaction only in the context of more frequent sex, and this association did not differ for same- and different-sex couples. On the other hand, extrinsic sexual motives (e.g., to please spouse) were associated with lower levels of sexual satisfaction in the context of high-frequency sex only among men married to men and women married to men, wherein the association was stronger for heterosexual couples compared to same-sex couples. These associations were not significant with less frequent sex. Results suggest that while frequent engagement in sex with intrinsic sexual motives benefits middle-aged adults’ sexual satisfaction regardless of relationship type, the sexual satisfaction of individuals married to men is vulnerable to the negative consequences of engaging in sex due to external pressure. These findings highlight the importance of considering how sexual experiences of men and women in midlife same-sex marriages compare to those of different-sex marriages.

